# A Novel Moisture Adjusted Vegetation Index (MAVI) to Reduce Background Reflectance and Topographical Effects on LAI Retrieval

**DOI:** 10.1371/journal.pone.0102560

**Published:** 2014-07-15

**Authors:** Gaolong Zhu, Weimin Ju, J. M. Chen, Yibo Liu

**Affiliations:** 1 Department of Geography, Minjiang University, Fuzhou, China; 2 International Institute for Earth System Sciences, Nanjing University, Nanjing, China; 3 Institute for Climate and Global Change Research, Nanjing University, Nanjing, China; 4 Department of Geography, University of Toronto, Toronto, Ontario, Canada; 5 Jiangsu Key Laboratory of Agricultural Meteorology, College of Applied Meteorology, Nanjing University of Information Science and Technology, Nanjing, China; University of New England, Australia

## Abstract

A new moisture adjusted vegetation index (MAVI) is proposed using the red, near infrared, and shortwave infrared (SWIR) reflectance in band-ratio form in this paper. The effectiveness of MAVI in retrieving leaf area index (LAI) is investigated using Landsat-5 data and field LAI measurements in two forest and two grassland areas. The ability of MAVI to retrieve forest LAI under different background conditions is further evaluated using canopy reflectance of Jack Pine and Black Spruce forests simulated by the 4-Scale model. Compared with several commonly used two-band vegetation index, such as normalized difference vegetation index, soil adjusted vegetation index, modified soil adjusted vegetation index, optimized soil adjusted vegetation index, MAVI is a better predictor of LAI, on average, which can explain 70% of variations of LAI in the four study areas. Similar to other SWIR-related three-band vegetation index, such as modified normalized difference vegetation index (MNDVI) and reduced simple ratio (RSR), MAVI is able to reduce the background reflectance effects on forest canopy LAI retrieval. MAVI is more suitable for retrieving LAI than RSR and MNDVI, because it avoids the difficulty in properly determining the maximum and minimum SWIR values required in RSR and MNDVI, which improves the robustness of MAVI in retrieving LAI of different land cover types. Moreover, MAVI is expressed as ratios between different spectral bands, greatly reducing the noise caused by topographical variations, which makes it more suitable for applications in mountainous area.

## Introduction

In recent decades, numerous spectral vegetation indices (VIs) derived from remotely sensed data have been developed to monitor the Earth’s vegetation cover and retrieve vegetation parameters such as leaf area index (LAI), fractional vegetation cover, biomass, and photosynthetic activity [1]–[4]. These VIs are often algebraic combinations of spectral reflectance in the red and near infrared (NIR) wavebands, for example, the most commonly used simple ratio (SR) [5] and normalized difference vegetation index (NDVI) [6]. They have been proved to be better than a single spectral band alone for estimating biophysical parameters of vegetation. However, the effectiveness of their applications is limited to different degrees by the effect of perturbing factors such as atmospheric conditions, topography, illumination and viewing geometry, sensor calibration, and soil background [7]–[8]. In forest ecosystems, the background often refers to all materials below the tree canopy (overstorey), including understorey (grass, shrub), moss, litter, and soil [9].

Varieties of VIs have been developed to reduce part of noise caused by these perturbing factors. Based on the so-called soil line concept [10], several soil adjusted VIs have been proposed to correct the perturbation of soil background, such as PVI [11], SAVI [12], WDVI [2], TSAVI [13], MSAVI [14], OSAVI [8], and GESAVI [15] (see Table 1). These two-band SAVI family indices appear to be less sensitive to soil brightness changes, but are more applicable to retrieving biophysical parameters of vegetation with relatively homogeneous canopies such as grasslands and croplands [7]–[8]. In order to enhance the sensitivity to vegetation change and minimize the noise caused by other factors, some three-band vegetation indices were developed by incorporating the blue or shortwave infrared (SWIR) bands. For instance, the atmospherically resistant vegetation index (ARVI) was designed to minimize atmospheric noise [16], and enhanced vegetation index (EVI) can reduce both the effects of atmospheric condition and soil background [17]. The modified normalized difference vegetation index (MNDVI) [18] and reduced simple ratio (RSR) [19] which combine the reflectance in the red, NIR, and SWIR bands are able to reduce the background effects. Although these indices might have some advantages for specific purposes, they are not based on the band ratio form and much of the noise may be retained or even enhanced [20]–[21]. It has been recognized that taking ratios between different spectral bands has the advantage of reducing unwanted noise caused by topography because the topographic effects often make the reflectances in the different bands change in similar proportions in the same direction. Therefore, efforts should be made to develop three-band index in band-ratio form.

**Table 1:** Formulas of several commonly used vegetation indices.

Vegetation index	Formula	Reference
Simple ratio (SR)	*SR* = *NIR*/*R*	[5]
Normalized difference vegetation index (NDVI)	*NDVI* = (*NIR*–*R*)/(*NIR*+*R*)	[6]
Perpendicular vegetation index (PVI)	*PVI* = (*NIR*-*aR*-*b*)/(*a* ^2^+1)^1/2^	[11]
Soil adjusted vegetation index (SAVI)	*SAVI* = (*NIR*–*R*)/(*NIR*+*R*+*L*)(1+*L*)	[12]
Weighted difference vegetation index (WDVI)	*WDVI* = *NIR*-*aR*	[2]
Transformed soil adjusted vegetation index (TSAVI)	*TSAVI* = *a*(*NIR*-*aR*-*b*)/(*R*+*a*(*NIR*-*b*)+*X*(1+*a* ^2^))	[13]
Modified soil adjusted vegetation index (MSAVI)	*MSAVI* = (2*NIR*+1–((2*NIR*+1)^2^–8(*NIR*–*R*))^1/2^)/2	[14]
Optimized soil adjusted vegetation index (OSAVI)	*OSAVI* = (*NIR*–*R*)/(*NIR*+*R*+*Y*)	[8]
Generalized soil adjusted vegetation index (GESAVI)	*GESAVI* = (*NIR*–*aR*–*b*)/(*R*+*Z*)	[15]
Atmospherically resistant vegetation index (ARVI)	*ARVI* = (*NIR-RB*)/(*NIR+RB*), *RB* = *R*-*γ*(*B*–*R*)	[16]
Modified normalized difference vegetation index (MNDVI)	*MNDVI* = *NDVI*×(*SWIR_max_*–*SWIR*)/(*SWIR_max_*–*SWIR_min_*)	[18]
Enhanced vegetation index (EVI)	*EVI* = 2.5×((*NIR*–*R*)/(*NIR*+*6R*−7.5*B*+1))	[17]
Reduced simple ratio (RSR)	*RSR* = *SR*×(*SWIR_max_*–*SWIR*)/(*SWIR_max_*–*SWIR_min_*)	[19]
Moisture adjusted vegetation index (MAVI)	*MAVI* = (*NIR*–*R*)/(*NIR*+*R*+*SWIR*)	This paper

Note: *B, R*, *NIR*, and *SWIR* are the surface reflectance in the blue, red, near infrared, and shortwave infrared bands, respectively. *SWIR_max_* and *SWIR_min_* are the maximum and minimum surface reflectance in the SWIR band, respectively. *SWIRmax* and *SWIRmin* are defined as the 1% minimum and maximum cutoff points in the histogram of the SWIR band reflectance here. *a* and *b* are the slope and intercept of the soil line, respectively. *L*, *X*, *Y*, and *Z* are soil background adjusted factors. *γ* is an atmospheric self-correcting factor which depends on aerosol types.

In this study, we develop a new three-band VI in band-ratio form, namely a moisture adjusted vegetation index (MAVI) (see Table 1). The sensitivity of MAVI to LAI is evaluated using LAI measured in two forest and two grassland areas and Landsat-5 data. Furthermore, the background reflectance effects of MAVI on forest canopy LAI retrieval are also investigated using the canopy reflectance of Jack Pine and Black Spruce forests with different background conditions simulated by the 4-Scale model [22]. Finally, we investigate how MAVI responds to topographical variations relative to other VIs and whether this ratio principle helps reduce topographical influence on VIs.

## Materials and Methods

### Ethics Statement

Field LAI measurements were performed in Tiantongshan Mountain forest, Maoershan Mountain forest, Hulunbeier grassland, and Xinlinhaote grassland (described in Section 2.2 and Section 2.3). The ground measurement permits were issued by Tiantongshan Forest Ecosystem Observation and Research Station in Zhejiang Province, Maoershan Forest Ecosystem Observation and Research Station in Heilongjiang Province, Hulunbeier Grassland Ecosystem Observation and Research Station of the Ministry of Agriculture, and Inner Mongolia Grassland Ecosystem Research Station the Chinese Academy of Sciences, respectively.

### 2.1 Design of MAVI

The SWIR band within the 1.5–1.75 µm range provides valuable complementary information relative to visible and NIR, regarding the geometrical structure of the canopy and on the optical properties of the underlying soil [23]. SWIR reflectance is strongly related to the canopy-equivalent water thickness, which provides a possibility of inferring canopy closure from remotely sensed data [24]. Panigrahy and Parihar proved that classification accuracy of crops was significantly improved by incorporating SWIR reflectance [25]. This band has been used to develop vegetation indices together with red and NIR bands in many previous studies [26]–[27]. Nemani et al. and Brown et al. used SWIR reflectance to modify NDVI and SR, resulting in a decreased sensitivity to background noise while improving their correlations with LAI [18]–[19]. Lymburner et al. indicated that the specific leaf area vegetation index (*SLAVI* = *NIR*/(*R*+*SWIR*)) had a strong positive correlation with specific leaf area index [28].

Because SWIR reflectance is sensitive to water content in the canopy as the canopy liquid water absorption is linearly related to LAI [29], a new vegetation index incorporating SWIR reflectance for improving LAI retrieval, namely the moisture adjusted vegetation index (MAVI) is developed empirically as:
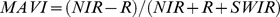
(1)which can be rewritten as



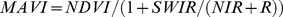
(2)


In general, an increase in the vegetation amount (LAI) causes a decrease in the SWIR and red reflectance and an increase in the NIR reflectance and NDVI. Because the absolute amount of the change in the red reflectance is usually less than that in the NIR and SWIR reflectance, the value of 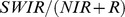 in Equation (2) decreases as the LAI increases. Consequently, the numerator and denominator in Equation (2) show an inverse relationship when the vegetation amount changes. This indicates MAVI potentially has higher sensitivity to LAI than NDVI. MAVI is expressed as ratios between the reflectance of the three bands, which gives it the potential to minimize the influences of external perturbing factors, such as the changes of illumination and observation geometry, complex topography, and instrument calibration defects.

### 2.2 Study Areas

**Figure 1:**
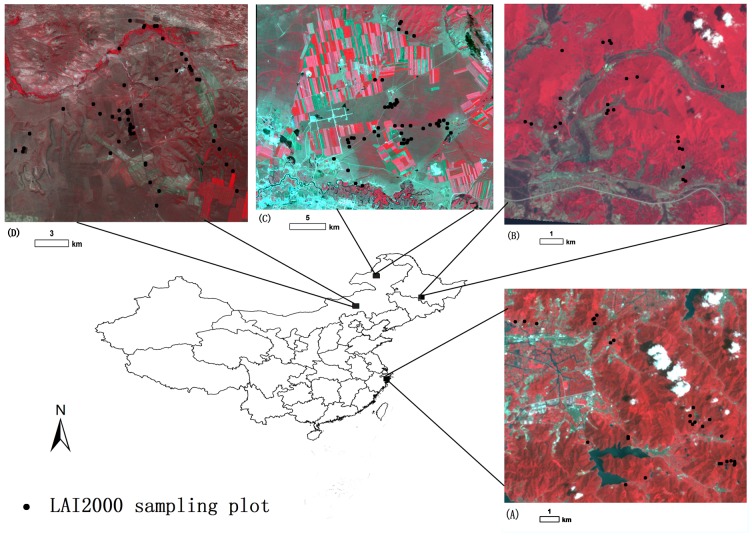
Areas in which LAI was measured. The effectiveness of the newly developed moisture adjusted vegetation index (MAVI) is tested using LAI measured in two grassland and two forest study areas in China: (A) Tiantongshan Mountain forest, (B) Maoershan Mountain forest, (C) Hulunbeier grassland, (D) Xinlinhaote grassland. The images composed of reflectance in bands 4, 3 and 2 from Landsat-5 TM are also shown.

The effectiveness of MAVI is tested using LAI measured in two grassland and two forest areas (Figure 1). Table 2 summarizes the major characteristics of these four areas. Tiantongshan Mountain lies in Zhejiang Province of eastern China. The study area is located in the subtropical monsoon climate zone with a mean annual temperature of 16.2°C and mean annual precipitation of 1374.7 mm [30]. The elevations are mostly below 500 m. The forests consist of evergreen and deciduous broadleaf species and coniferous species. Maoershan Mountain is located in Heilongjiang Province of northern China. It covers an area of 266.2 km^2^. The mean annual temperature and precipitation in this area are 2.8°C and 723.8 mm, respectively [31]. The elevation in this hilly highland varies from 250 m to 817 m with a mean elevation of 428 m. It is covered by a regenerative forest with various types, including broadleaf, needle-leaf, and mixed forest. The forests are approximately 50 years old.

**Table 2:** The information on the study areas and the Landsat-5 TM images used in this study.

Study areas	Latitude range	Longitude range	Vegetationtype	Field dates	No. of Plots	LAI range	TM scene date	Path/row
Tiantongshan	29.7755°–29.8553°N	121.6956°–121.8103°E	forest	19–25 Sep., 2009	23	3.41–6.66	18 Aug., 2009	118/39
Maoershan	45.2659°–45.3232°N	127.4957°–127.6047°E	forest	12–20 Jul., 2009	23	1.61–6.55	24 Jun., 2009	117/28
Hulunbeier	49.2744°–49.4772°N	119.9148°–120.1401°E	grassland	21–26 Jun., 2010	52	0.46–4.06	21 Jun., 2010	123/26
Xilinhaote	43.5025°–43.631°N	116.5456°–116.7898°E	grassland	29 Jun.–4 Jul., 2010	51	0.65–4.7	21 Jun., 2010	123/30

The two grassland areas are near Hulunbeier City and Xilinhaote City of the Inner Mongolian Plateau. The elevation in Hulunbeier grassland varies from 650 m to 700 m. The mean annual temperature varies from −2.0°C to −1.0°C, and annual precipitation ranges from 350 mm to 400 mm [32]. It is a representative meadow steppe ecosystem with the height of the grass ranging from 0.3 m to 0.5 m. The average elevation in the Xilinhaote grassland is approximately 1100 m. The Xilinhaote grassland belongs to the semiarid grassland climate region with a mean annual temperature of 2.0°C and mean annual precipitation of about 350 mm, mainly occurring from June to August [33]. This site is a typical steppe ecosystem in northern China.

### 2.3 LAI Measurement

Field LAI measurements at Maoershan and Tiantongshan were carried out from 12 to 20 July and from 19 to 25 September, 2009, respectively. Each area had 23 plots in needle-leaf forests, broadleaf forests, and mixed forests which represent major vegetation species in the study areas. These plots were located on relatively flat terrains and composed of relatively homogeneous vegetation species. Two LAI-2000 (Li-Cor, Nebraska, USA) units were used to measure LAI. One unit was set up at an open location close to the observation plots to acquire *A* readings (above-canopy) at 30 s intervals. The other one was used to record *B* readings below the canopy at each sampling plot. A 270° view cap was used on each of LAI-2000 units to avoid the influence of the operator on the sensor. At each sampling plot, two 50 m parallel transects separated by 25 m were laid in the centre of the plot. Along each transect, measurements were made just above the ground level at 6 equally spaced spots using LAI-2000. At each spot, two *B* readings were made at random locations within a 1 m diameter circle. For each sampling spot, LAI was calculated using the *A* and *B* readings acquired simultaneously. Because of the relatively severe multiple scattering effect of blue wavelength on the data of the fifth ring of LAI-2000, only the data of rings1–4 was used to calculate LAI. The LAI value for a plot was computed as the mean of 24 LAI measurements (2 LAI/spot×6 spot/transect×2 transect). LAI measurements were conducted when the sky was overcast or in hours just after sunset. The values of measured LAI in Maoershan and Tiantongshan range from 1.61 to 6.55 and from 3.41 to 6.66, respectively.

The LAI in Hulunbeier and Xilinhaote grasslands was measured from 21 to 26 June and from 29 June to 4 July, 2010, respectively. In the study areas of Xilinhaote and Hulunbeier, 51 and 52 plots were set up, respectively. Two 40 m parallel transects were placed at a distance of 25 m in each plot. Five sampling spots were spaced in 10 m intervals along each transect. A narrow groove about 0.05 m deep was created at each sampling spot to allow the upper surface of the LAI-2000 sensor head to be placed at the same level as the ground surface to measure LAI of the short grass. At each plot, the mean LAI value was obtained from 12 LAI-2000 readings arranged in a sequence: starting with one *A* reading (above-grass reading), followed by 10 *B* readings (below-grass reading in each groove), and ended with another *A* reading. Once again the 270° view cap was used to avoid operator interference with the sensor. All measurements were made near sunrise, sunset, or when overcast. The LAI of Hulunbeier and Xilinhaote grasslands is shown to vary from 0.46 to 4.06 and from 0.65 to 4.7, respectively. A GPS device was used to survey the geographical position of the centre of each plot in these four study areas.

### 2.4 Remotely Sensed Data Processing

Four Landsat-5 Thematic Mapper (TM) scenes covering these four study areas were downloaded from United States Geological Survey (USGS) (Table 2). These images were registered to within half a pixel with ground control points recorded in the field campaign. Radiometric correction was made using the gain and offset parameters of each band included in the Landsat-5 TM header files. Surface reflectance was obtained after atmospheric correction using the 6S code [34], with inputs of a continental air-mass, mid-latitude summer, a uniform target, and 40 km atmospheric visibility. Surface reflectance images were projected to the UTM/WGS84 coordinate system with a 30 m spatial resolution. The LAI sampling plots were positioned on the surface reflectance images using latitude/longitude coordinates measured by GPS. Because the plots were approximately 50 m×50 m and the pixel size of TM image was 30 m, the average surface reflectance in red, NIR, and SWIR bands was extracted from a 3 by 3 pixels window centered on each plot. The average surface reflectance in bands 3, 4 and 5 from Landsat-5 TM of each plot was used to calculate VIs.

### 2.5 VI-LAI Modeling

The semi-empirical exponential function based on the modified Beer’s law is used to fit VI-LAI relationships:
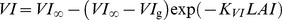
(3)where *VI*_∞_ is the asymptotic value of a specific VI when LAI reaches infinity (in fact, this limiting value is always reached when LAI approaches 8); *VI*_g_ is the VI value of the bare soil (*LAI* = 0). The difference between *VI*_∞_ and *VI*_g_ controls the dynamic range of the VI. *K_VI_* is an extinction coefficient determining the sensitivity of VI to LAI.

The Marquardt-Levenberg algorithm [35] is used to determine the optimal fitting parameters of each selected VI (NDVI, SAVI, OSAVI, MSAVI, RSR, MNDVI, and MAVI, see Table 1) in the four study areas in terms of the largest adjusted coefficient of determination (*R* ^2^) of each VI-LAI relationship. For each VI, these fitted parameters depend on illumination and viewing geometries, soil optical properties, and leaf inclination [13], [36]. The performance of MAVI is assessed through comparing its ability to predict LAI with the abilities of other VIs to predict LAI. The soil adjusted factors of these VIs (Table 1) are set as the values recommended by their authors (*L* = 0.5, *X* = 0.08, *Y* = 0.16, and *Z* = 0.35).

### 2.6 Assessing Sensitivity of VIs to Forest Background Reflectance

Forest background reflectance has an important influence on the accuracy of canopy LAI estimation in the case of low to intermediate canopy cover [9]. The canopy reflectance of Jack Pine and Black Spruce forests with different background conditions simulated by Brown et al. [19] is used here to investigate the sensitivity of VIs to background disturbances. They employed the 4-Scale model [22] to simulate the canopy reflectance of Jack Pine and Black Spruce forests with different types of backgrounds. The in situ spectroradiometric measurements of the backgrounds, including lichen, sphagnum moss, and forest soils, taken in the two sites are used in the simulations of canopy reflectance. A synthetic background of 50% of water and 50% of moss spectra, representing an extreme case, is also used to simulate the canopy reflectance of Black Spruce forest. In the simulations, the canopy LAI of Jack Pine and Black Spruce forests is allowed to change from 0.5 to 6 at an interval of 0.5 to analyze the change in the sensitivity of VIs to background reflectance with canopy density. A complete description of these modeled data can be found in Brown et al. [19].

The sensitivity of VIs to background reflectance (*T_VI_*) can be characterized as [37]:
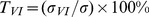
(4)where *σ_VI_* refers to the standard deviation of VI values corresponding to a given LAI value and *σ* is the standard deviation of the VI over the whole range of measured LAI. A smaller *T_VI_* value indicates higher efficiency and low sensitivity to background reflectance for a VI.

### 2.7 Assessing Topographical Effects on VIs

The study area in Maoershan Mountain is selected to assess the topographical effects on VIs. It is a low elevation highland with a mean slope of 14.2°. As the altitude increases, the slope and forest age increase slowly due to relatively frequent deforestation at flat terrains and lower altitudes. In assessing topographical effects on VIs, pixels with slopes from 3° to 27° are binned into 5-degree slope intervals. The pixels with slopes more than 27° are excluded since they are too few in the study area. The five classes are tagged by the median value of the slope range of each class hereafter (i.e., 5°, 10°, 15°, 20°, 25°). In each class, the mean values at every aspect angle of the selected VI are calculated. The study area is covered by high density forests with almost closed canopies, so the effects of soil background on VIs are negligible. Because the study area is relatively small, the atmospheric conditions of each pixel can be assumed identical, and as the study area is composed of relatively homogeneous vegetation species, the variations of a VI within each slope class are assumed a result of topography.

The coefficient of variation (*CV*) is used to evaluate the effects on VIs resulting from topography variation. It is defined as,
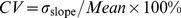
(5)where *σ*_slope_ and *Mean* are the standard deviation and the mean value of VI corresponding to a given slope class, respectively. The *CV* value represents the noise caused by topographic variations. A smaller *CV* value indicates a smaller topographic effect.

As the second criterion, the topographic effects on VIs can also be expressed in the following way:
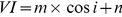
(6)

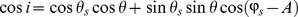
(7)where *i* is the incidence angle defined as the angle between the direction of the sun and the local surface normal, which represents one of the most important perturbations in remote sensing over mountainous terrain [38]. *m* and *n* represent the slope and intercept of the linear regression line between *VI* and cos*i*; *θ_s_* and *φ_s_* are the solar zenith angle and the solar azimuth angle, respectively; *θ* and *A* are the slope and aspect of an inclined pixel, respectively. These are derived from the 30 m DEM of the study area. The coefficient of determination (*R* ^2^) of the regression represents the sensitivity of VI to topographic variations. The *R* ^2^ value increases as the topographic effect increases.

## Results and Discussions

### 3.1 Relationships between VIs and LAI

**Figure 2:**
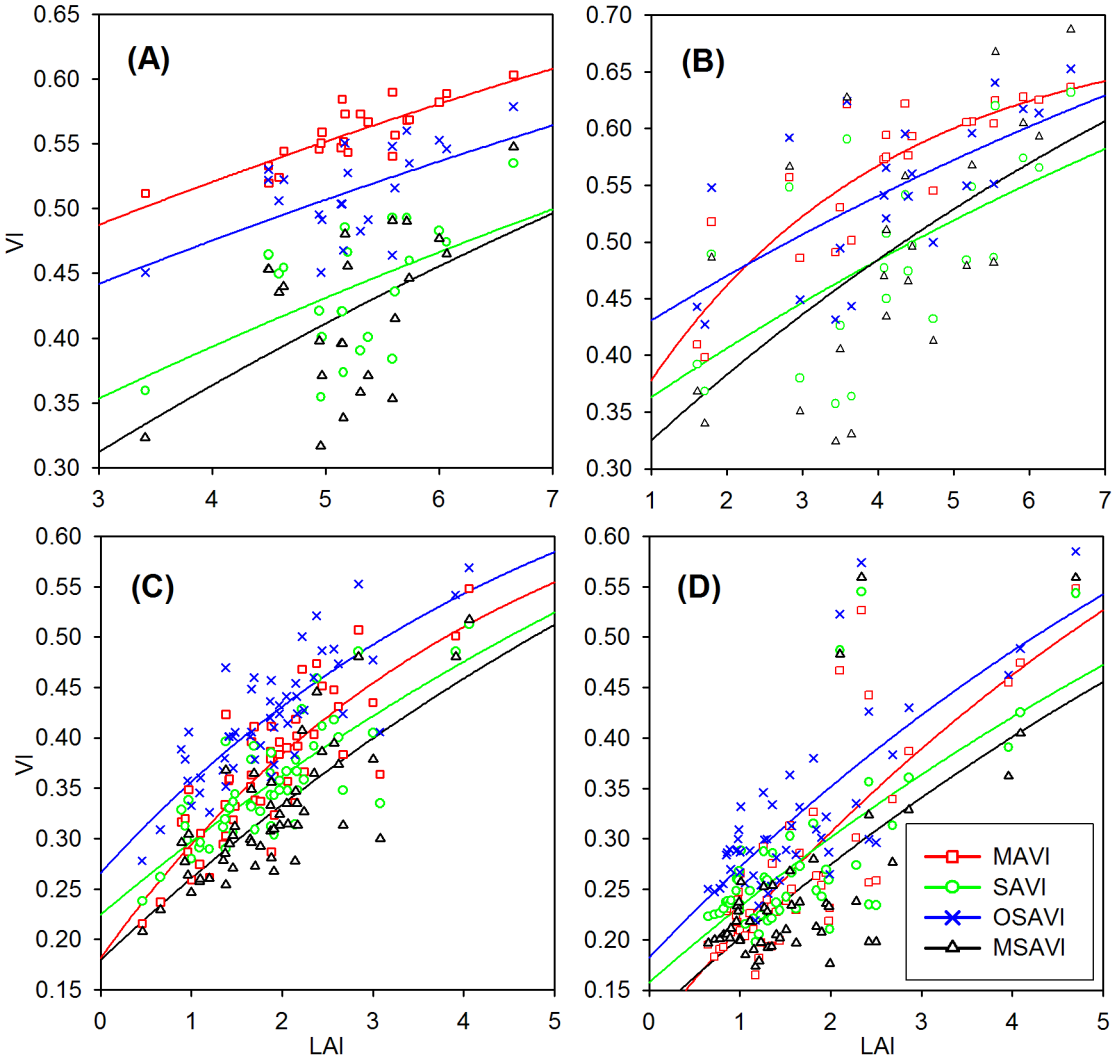
The best fitted relationships between LAI and vegetation indices. The MAVI and three soil adjusted vegetation indices (SAVI, OSAVI, and MSAVI) are compared in the four study areas: (A) Tiantongshan, (B) Maoershan, (C) Hulunbeier, and (D) Xilinhaote. The statistics of the best fitted VI-LAI relationships are listed in Table 3. MAVI produces a higher *R* ^2^, smaller normalized RMSE of retrieved LAI compared with the three soil adjusted vegetation index in both forest and grassland areas.

**Figure 3:**
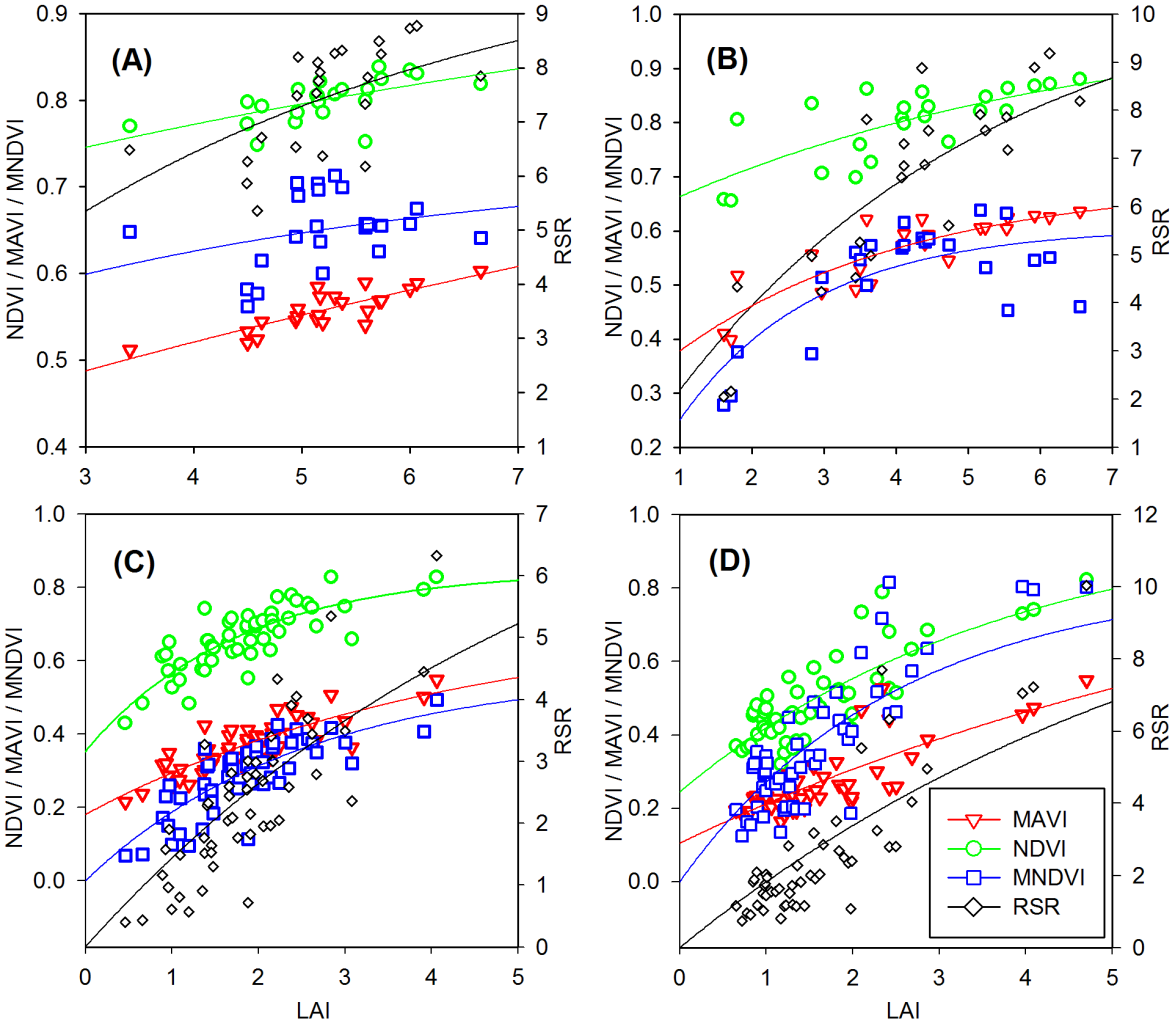
The best fitted relationships between LAI and vegetation indices. The MAVI and three selected vegetation indices (NDVI, MNDVI, and RSR) are compared in the four study areas: (A) Tiantongshan, (B) Maoershan, (C) Hulunbeier, and (D) Xilinhaote. MAVI produces the largest *R* ^2^ and the smallest normalized RMSE of estimated LAI in Tiantongshan and Hulunbeier. The performance of MAVI based on *R* ^2^ and RMSE is only slightly second to RSR in Maoershan and Xilinhaote. These results prove that MAVI has stable correlations with LAI under different cover types through incorporating the SWIR reflectance in band-ratio form.

Seven VIs including NDVI, SAVI, OSAVI, MSAVI, RSR, MNDVI, and MAVI, full descriptions found in Table 1, are selected and calculated from the TM surface reflectance image to investigate their sensitivity to LAI. Figure 2 and Figure 3 show the best fitted relationships between the selected VIs and LAI in the four study areas. All VIs are almost linearly correlated with LAI in the Tiantongshan, in which vegetation density is relatively high and in a smaller range compared with other areas. RSR, SAVI, OSAVI, and MSAVI also show approximately linear correlations with LAI in the remaining three study areas. NDVI, MNDVI and MAVI increase rapidly with increasing LAI at low LAI values, and increase slowly with increasing LAI at high LAI values.

Table 3 shows the best fitted relationships between the selected VIs and LAI in the four study areas. In order to facilitate the comparison, the RMSE for each VI is normalized by the difference between the maximum and minimum value of each VI corresponding to the whole range of measured LAI. The fitted parameters in the VI-LAI relationships vary considerably with VIs and cover types. The dynamic ranges (VI_∞_-VI_g_) of these selected VIs are quite different in the four study areas. This is particularly true in Xilinhaote where the dynamic range is relatively larger. Among these two-band VIs, MSAVI has the largest dynamic range values of 0.8693, 0.7391, 0.8206, and 0.8784 in Tiantongshan, Maoershan, Hulunbeier, and Xinlinhaote, respectively, while NDVI has the smallest dynamic range values of 0.354, 0.4002, 0.4956, and 0.7552 in these areas. These results are in agreement with the previous findings of Wu et al. [36]. NDVI presents *R* ^2^ values of 0.3703, 0.5338, 0.6667, and 0.6647 in the four study areas, which are larger than those of the three soil adjusted VIs (SAVI, OSAVI, and MSAVI), and has smaller normalized RMSE of 0.2278, 0.2171, and 0.1415 in Tiantongshan, Maoershan, and Xinlinhaote, respectively, indicating this ratio-based simple vegetation index has some advantages for retrieval of LAI over more complex VIs. OSAVI shows better efficiency than SAVI and MSAVI in all study areas. The three soil adjusted VIs all present stronger correlations with LAI in grassland areas than seen in forest areas, suggesting they are more applicable for retrieving LAI for vegetations with relatively homogeneous canopies such as grasslands and croplands than for vegetations with distinct canopy structure such as forests.

**Table 3:** The best fitted VI-LAI relationships and their statistics.

Study areas	VI	*VI* = *VI*_∞_−(*VI*_∞_−*VI*_g_)exp(−*K_VI_LAI*)	STDV	(*VI*_max_−*VI*_min_)	*R* ^2^	RMSE	Normalized RMSE
Tiantongshan	NDVI	*y* = 1–0.354exp(−0.11*x*)	0.025	0.090	0.3703	0.0205	0.2278
	SAVI	*y* = 1–0.7837exp(−0.064*x*)	0.048	0.180	0.2600	0.0431	0.2389
	OSAVI	*y* = 1–0.672exp(−0.0619*x*)	0.035	0.128	0.3291	0.0305	0.2381
	MSAVI	*y* = 1–0.8693exp(−0.078*x*)	0.061	0.231	0.2571	0.0549	0.2380
	RSR	*y* = 10.5–10.5exp(−0.237*x*)	0.990	3.424	0.3648	0.8274	0.2417
	MNDVI	*y* = 0.7299–0.2589exp(−0.2271*x*)	0.042	0.151	0.0879	0.0425	0.2806
	MAVI	*y* = 1–0.6268exp(−0.067*x*)	0.024	0.091	**0.6975**	0.0137	**0.1501**
Maoershan	NDVI	*y* = 1–0.4002exp(−0.1724*x*)	0.068	0.224	0.5338	0.0487	0.2171
	SAVI	*y* = 1–0.6835exp(−0.0703*x*)	0.082	0.275	0.3902	0.0674	0.2454
	OSAVI	*y* = 1–0.6115exp(−0.0715*x*)	0.070	0.225	0.4409	0.0546	0.2423
	MSAVI	*y* = 1–0.7391exp(−0.0901*x*)	0.108	0.363	0.3802	0.0893	0.2459
	RSR	*y* = 11–11exp(−0.222*x*)	2.022	7.143	**0.7580**	1.0435	**0.1461**
	MNDVI	*y* = 0.6052–0.6052exp(−0.5351*x*)	0.101	0.360	0.6281	0.0644	0.1789
	MAVI	*y* = 0.6904–0.4266exp(−0.3107*x*)	0.068	0.238	**0.7417**	0.0361	**0.1517**
Hulunbeier	NDVI	*y* = 0.8482–0.4956exp(−0.5686*x*)	0.086	0.398	0.6667	0.0505	0.1269
	SAVI	*y* = 1–0.7754exp(−0.0978*x*)	0.056	0.274	0.6496	0.0340	0.1239
	OSAVI	*y* = 0.776–0.5105exp(−0.196*x*)	0.060	0.291	0.6622	0.0357	0.1228
	MSAVI	*y* = 1–0.8206exp(−0.1041*x*)	0.063	0.309	0.6382	0.0389	0.1258
	RSR	*y* = 9–9exp(−0.1739*x*)	1.234	5.927	0.6386	0.7569	0.1277
	MNDVI	*y* = 0.5713–0.5713exp(−0.3985*x*)	0.100	0.424	0.6449	0.0607	0.1430
	MAVI	*y* = 0.7205–0.5394exp(−0.2354*x*)	0.069	0.332	**0.6924**	0.0391	**0.1176**
Xilinhaote	NDVI	*y* = 1–0.7552exp(−0.2631*x*)	0.120	0.503	**0.6647**	0.0712	**0.1415**
	SAVI	*y* = 1–0.8425exp(−0.0936*x*)	0.078	0.348	0.5071	0.0562	0.1615
	OSAVI	*y* = 1–0.8181exp(−0.1163*x*)	0.083	0.366	0.5923	0.0541	0.1479
	MSAVI	*y* = 1–0.8784exp(−0.0957*x*)	0.086	0.386	0.4741	0.0640	0.1658
	RSR	*y* = 12.5–12.5exp(−0.1579*x*)	1.995	9.297	**0.6669**	1.1749	**0.1264**
	MNDVI	*y* = 0.825–0.825exp(−0.4007*x*)	0.186	0.691	**0.6858**	0.1065	0.1541
	MAVI	*y* = 1–0.8952exp(−0.1275*x*)	0.092	0.384	**0.6627**	0.0544	**0.1418**

Note: STDV is the standard deviation of each VI corresponding to the whole range of measured LAI. (*VI*_max_–*VI*_min_) is the difference between the maximum and minimum value of each VI corresponding to the whole range of measured LAI. The last column gives the normalized RMSE (i.e., *RMSE*/(*VI*_max_–*VI*_min_)) for each VI. Values in bold font indicate high performance.

The three-band VIs (MNDVI, RSR, and MAVI) all perform significantly better than the two-band VIs in estimating LAI in Maoershan and Xinlinhaote. However, MNDVI and RSR are poorer predictors of LAI compared with two-band VIs in Tiantongshan and Hulunbeier, which may be due to their non-ratio forms and the difficulty in properly determining the values of SWIR_max_ and SWIR_min_ in them. Instead, MAVI produces the largest *R* ^2^ of 0.6975 and 0.6924 and the smallest normalized RMSE of 0.1501 and 0.1176 in Tiantongshan and Hulunbeier, respectively, indicating the superior performance of MAVI over other VIs in retrieving LAI. These results prove that MAVI has stable correlations with LAI under different cover types due to incorporating the SWIR reflectance in band-ratio form.

### 3.2 Sensitivity of VIs to Background Reflectance in Forest Canopy LAI Retrieval

**Figure 4:**
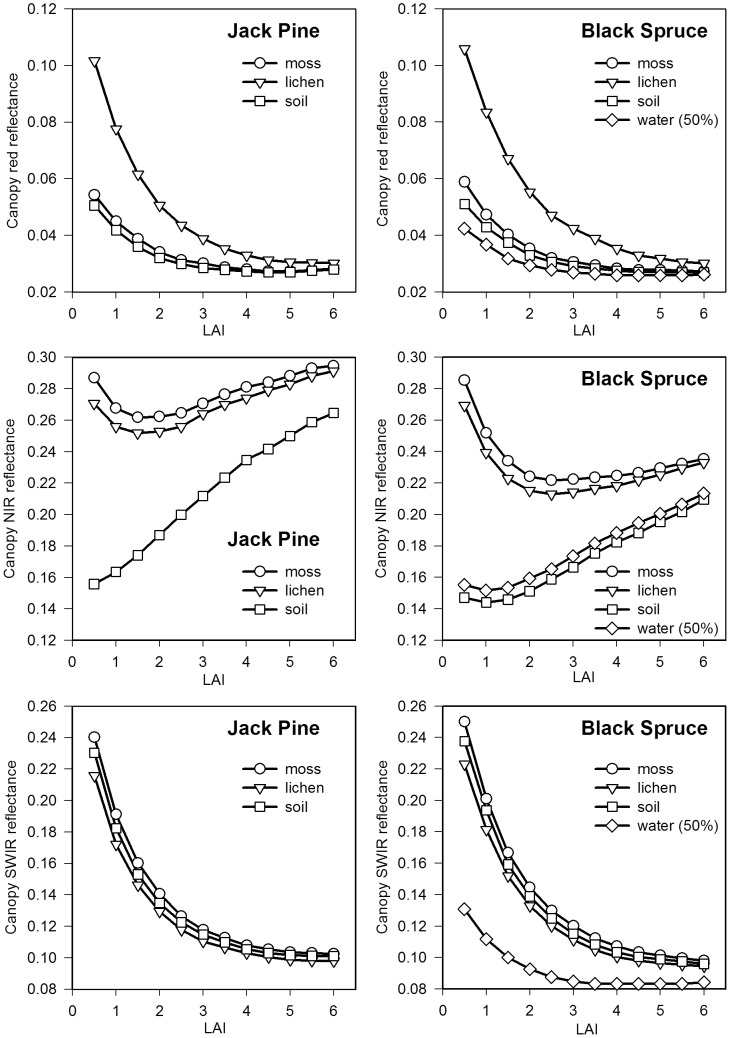
Canopy reflectance of Jack Pine and Black Spruce forests against LAI for different backgrounds. The canopy reflectance of Jack Pine and Black Spruce forests is simulated using the 4-Scale model against LAI for different backgrounds (moss, lichen, and forest soil). A synthetic background consisting of 50% of water and 50% of moss is also included for the Black Spruce forest [19]. The sensitivity of MAVI to background reflectance disturbances is investigated using these modeled results.

Figure 4 shows the canopy reflectance in the red, NIR, and SWIR bands simulated by the 4-Scale model for Jack Pine and Black Spruce forests with different background types. The red and SWIR reflectance decreases with increasing LAI for both Jack Pine and Black Spruce forests over the entire range of LAI. The canopy NIR reflectance shows different curves, depending on background types. At LAI values less than 2.0, the canopy NIR reflectance decreases with increasing LAI over the moss and lichen backgrounds due to the increase in the probability of observing shadowed background, while the canopy NIR reflectance increases slowly with increasing LAI at LAI values above 2. For non-vegetative backgrounds, the canopy NIR reflectance increases with increasing LAI over the entire range of LAI. The effects of different backgrounds on the canopy reflectance in the red, NIR and SWIR bands decrease with increasing LAI. The canopy SWIR reflectance presents an inverse curvilinear trend with increasing LAI and reaches an asymptote at an LAI of approximately 5, while the canopy red reflectance reaches an asymptote at an LAI of approximately 3. The similarity of SWIR reflectance across different backgrounds (lichen, moss and soil) compared to the range shown in the red and NIR reflectance, and the large sensitivity of SWIR to LAI are the main reasons for the better performance of RSR compared with SR in estimating canopy LAI [19]. This also demonstrates that the canopy SWIR reflectance can be used to quantify the vegetation amount as well as canopy closure in most cases [23]–[24].

**Figure 5:**
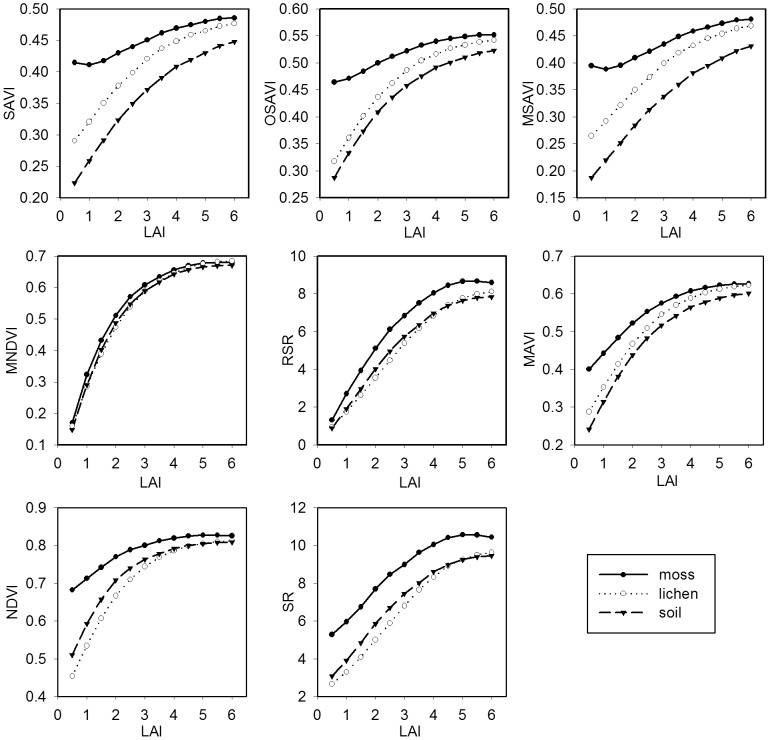
Background reflectance effects on vegetation indices at different LAI values in Jack Pine forest. The effects of different backgrounds (moss, lichen, and forest soil) on the selected vegetation indices (SAVI, OSAVI, MSAVI, MNDVI, RSR, MAVI, NDVI, and SR) are simulated using the 4-Scale model for the different LAI levels in Jack Pine forest. The forest background strongly affects the values of SAVI, OSAVI, MSAVI, and NDVI as the LAI values are less than 2. MAVI and SR can reduce the effects of forest backgrounds at low LAI values. RSR and MNDVI show the smallest background reflectance effects among these vegetation indices.

**Figure 6:**
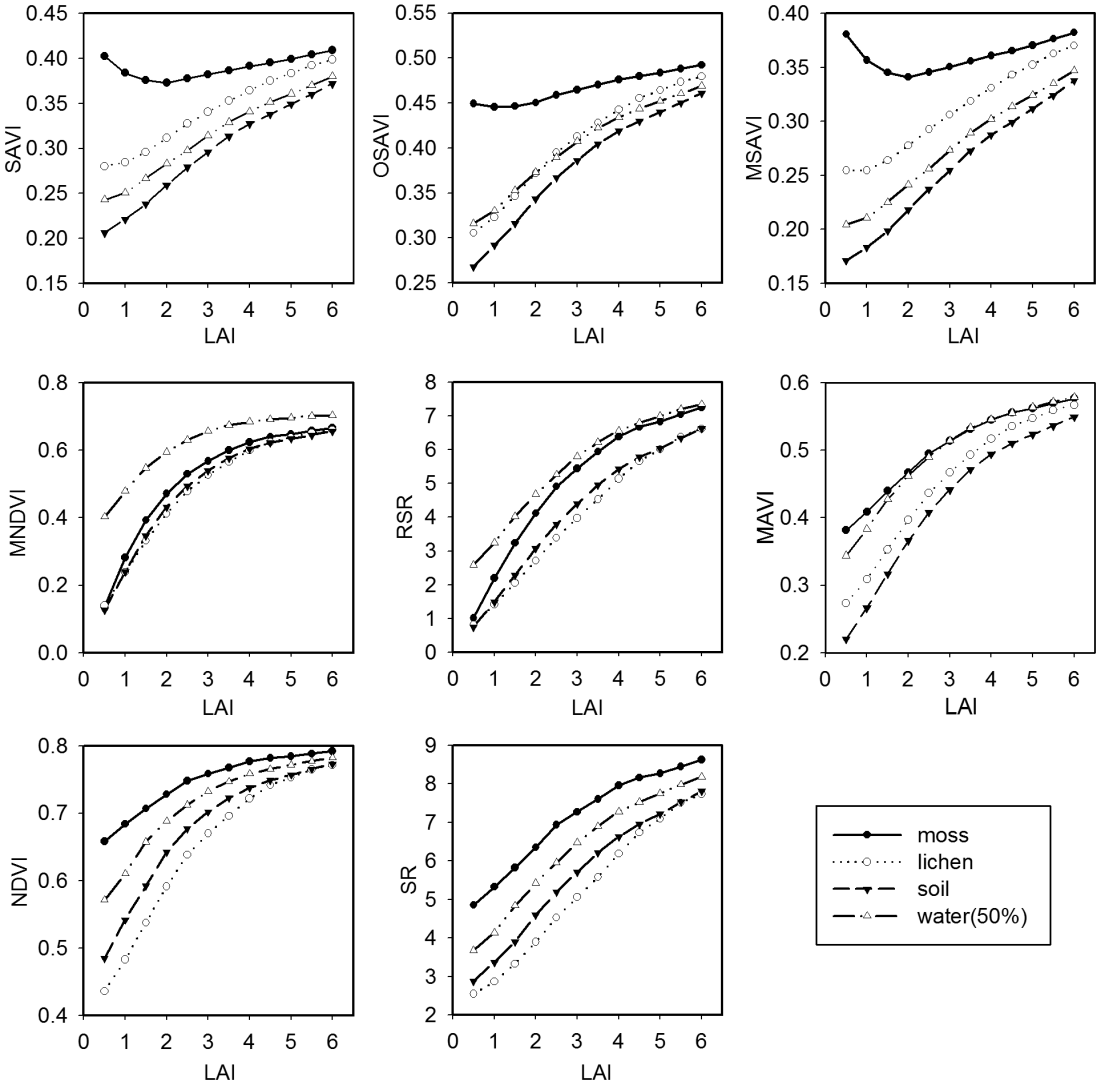
Background reflectance effects on vegetation indices at different LAI values in Black Spruce forest. The effects of different backgrounds (moss, lichen, forest soil, and the mixed background of water and moss) on the selected vegetation indices (SAVI, OSAVI, MSAVI, MNDVI, RSR, MAVI, NDVI, and SR) are simulated using the 4-Scale model for the different LAI levels in Black Spruce forest. The results are similar to those of Figure 5, but RSR and MNDVI do not perform much better than other vegetation indices in reducing the effect of the mixed background of water and moss.

**Figure 7:**
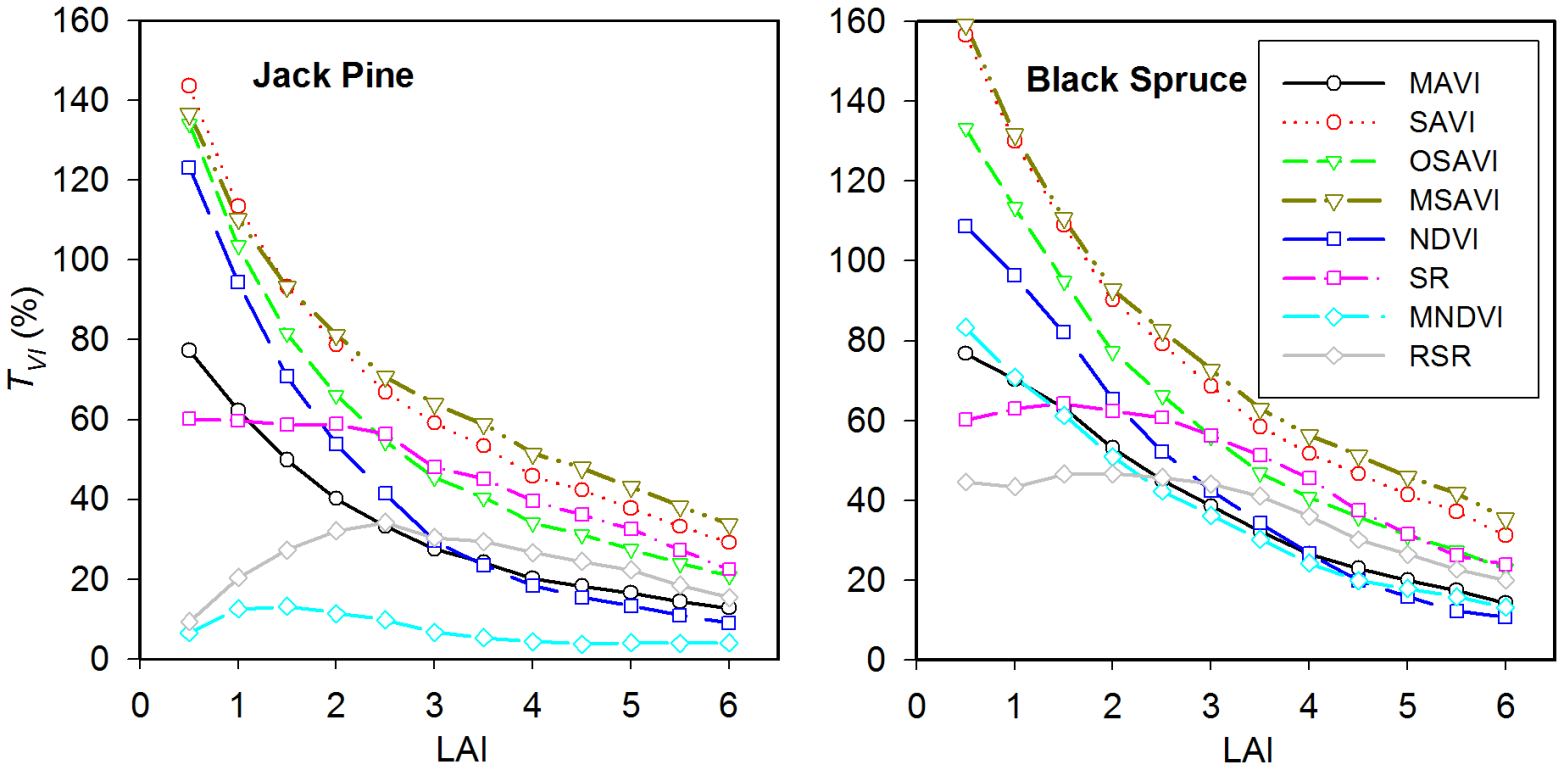
Sensitivity of different vegetation indices to forest background reflectance. The background reflectance strongly affects the values of SAVI, OSAVI, MSAVI, and NDVI in both Jack Pine and Black Spruce forests as the LAI values are less than 2, leading to larger *T_VI_* values of them compared with other vegetation indices. RSR and MNDVI present small *T_VI_* values at low LAI values, indicating that the background reflectance effects on them are smaller than other vegetation indices. MAVI has relatively small *T_VI_* values over the entire LAI ranges in both Jack Pine and Black Spruce forests. The results demonstrate that MAVI that combines the red, NIR and SWIR reflectance can reduce the effects of background reflectance on forest canopy LAI retrieval.

Figure 5 and Figure 6 illustrate the background reflectance effects on the calculated VIs at the different LAI levels in Jack Pine and Black Spruce forests. In general, the effects of different background reflectance on the canopy VIs decrease with increasing LAI for all VIs in both Jack Pine and Black Spruce forests, because the probability of observing background decreases with increasing LAI. The moss background strongly affects the values of SAVI, OSAVI, and MSAVI in both Jack Pine and Black Spruce forests as the LAI values are less than 2, leading to larger *T_VI_* values compared with other VIs (Figure 7). Similarly, the noise induced to NDVI by forest background reflectance shows large *T_VI_* values varying from 123% to 71% in Jack Pine forest and from 109% to 82% in Black Spruce forest corresponding to LAI from 0.5 to 1.5, which is in agreement with the conclusion of Nemani et al. that NDVI alone cannot be used to estimate LAI in open forest canopies [18]. SR presents relatively small *T_VI_* values changing from 60% to 59% in Jack Pine forest and from 60% to 64% in Black Spruce forest corresponding to LAI from 0.5 to 1.5, indicating that the background reflectance effects on SR are smaller than those on NDVI, which supports the statement by Chen and Cihlar that SR has the highest correlation with LAI in boreal forests [39]. RSR and MNDVI can largely reduce the effects of moss, lichen, and forest soil backgrounds on calculated VIs at low LAI values (Figure 5–7), mainly due to the fact that the SWIR reflectance in RSR and MNDVI can scale down the increased NIR response in open stands associated with understory vegetation or other highly reflective backgrounds [18]. However, RSR and MNDVI do not perform much better than other VIs in reducing the effect of the mixed background of water and moss since SWIR is sensitive to wet background leading to the relatively low SWIR reflectance (Figure 6). On the other hand, the efficiency of RSR and MNDVI to reduce background reflectance effects is strongly controlled by the maximum and minimum values of SWIR reflectance in them, which is difficult to determine properly in many practical applications.

MAVI has relatively small *T_VI_* values varying from 77% to 13% in Jack Pine forest and from 76% to 14% in Black Spruce forest over the entire LAI ranges (Figure 7). The results demonstrate that MAVI that combines the red, NIR and SWIR reflectance can reduce the effects of backgrounds on forest canopy LAI retrieval. Its underlying physical mechanism is similar to those of RSR and MNDVI, i.e., SWIR reflectance is highly sensitive to LAI at low LAI values and has the largest values in open forest canopies, which acts as an adjustment factor in the denominator of MAVI to moderate the increased NIR in open stands (Figure 4). However, the canopy SWIR reflectance is also sensitive to the wetness of background, especially at low LAI values. Fortunately, the wetness of background affects not only the canopy SWIR reflectance but also the canopy NIR and red reflectance (Figure 4). The ratio form of MAVI makes it possible to constrain the effects of the change in the wetness of background partially and to be suitable for retrieving LAI for forests with different types of backgrounds.

### 3.3 Topographic Effects on VIs

**Figure 8:**
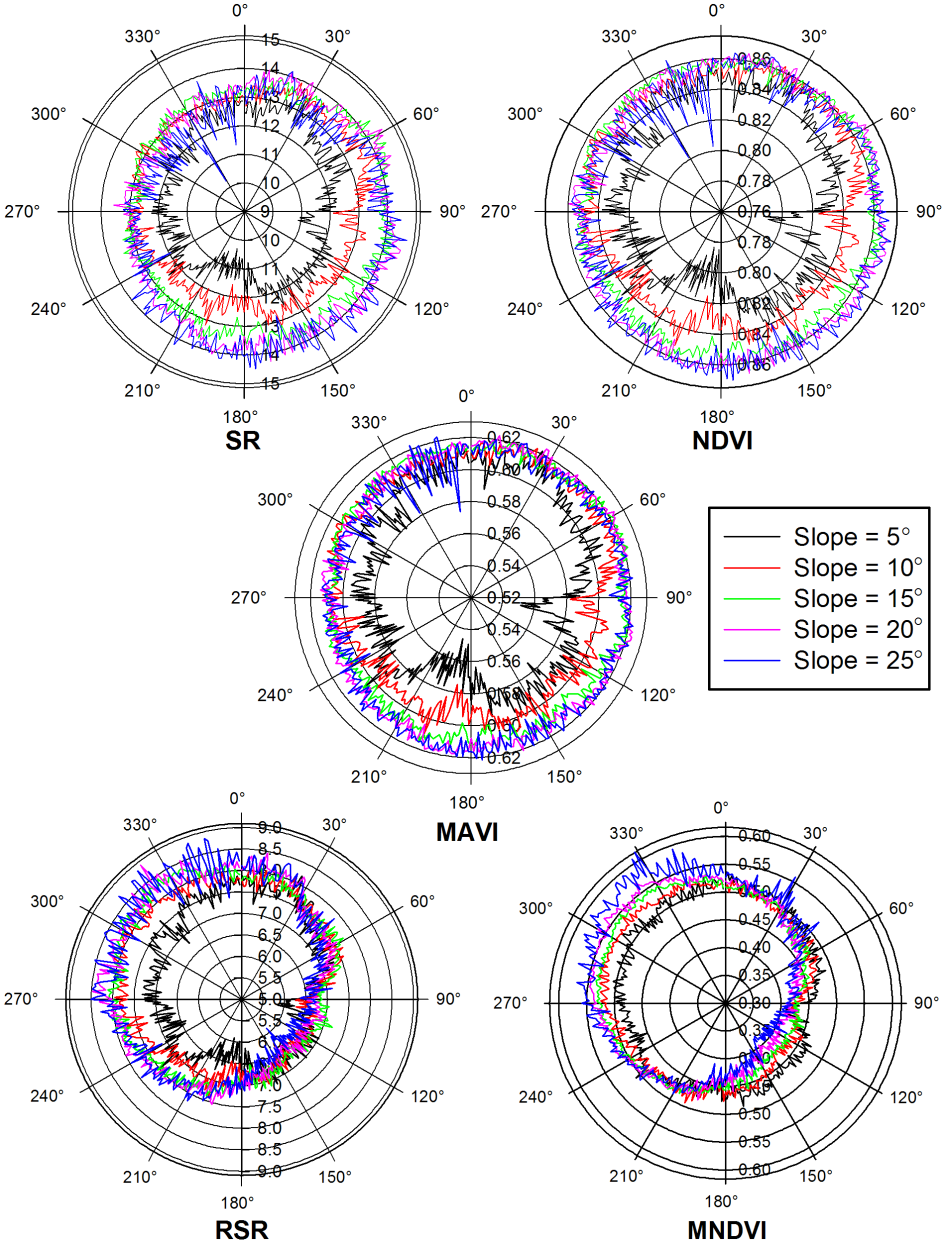
Variations of different vegetation indices with aspects in 5-degree slope intervals in Maoershan Mountain forest. The polar angle represents aspect, and the radius represents the mean values of each VIs at a given aspect on different slopes. Topography strongly affects RSR and MNDVI, resulting in negative biases on sun-facing slopes and positive biases on sun-backing slopes. The values of SR, NDVI, and MAVI increase as the slope increases, which is similar to the changes of forest age with slope. It can be inferred that vegetation indices expressed in band-ratio form are able to remove a large proportion of topographical noise.

Figure 8 shows the band-ratio VIs (SR, NDVI, and MAVI) and the non-band-ratio VIs (RSR and MNDVI) change with aspect at different slopes. When the slope is 5°, each VI varies greatly with aspect due to relatively large vegetation variations. When the slope is larger than 10°, the changes of SR, NDVI, and MAVI at a given slope are quite different from those of RSR and MNDVI. The values of SR, NDVI, and MAVI increase as the slope increases, which is similar to the changes of forest age with slope. However, the values of RSR and MNDVI decrease on sun-facing slopes and increase on sun-backing slopes as the slopes increase. At a given slope, the values of RSR and MNDVI of sun-facing slopes are much smaller than those of sun-backing slopes. The larger the slope, the larger the difference with regard to sun-facing and sun-backing slopes. Because forest ages are almost the same at all aspect according to the ground reference data, the values of each VI at a given slope should be independent of the aspect relative to the sun. Therefore, it can be inferred that VIs expressed in band-ratio form are able to remove a large proportion of topographical noise. Topography strongly affects RSR and MNDVI, resulting in negative biases on sun-facing slopes and positive biases on sun-backing slopes.

**Figure 9:**
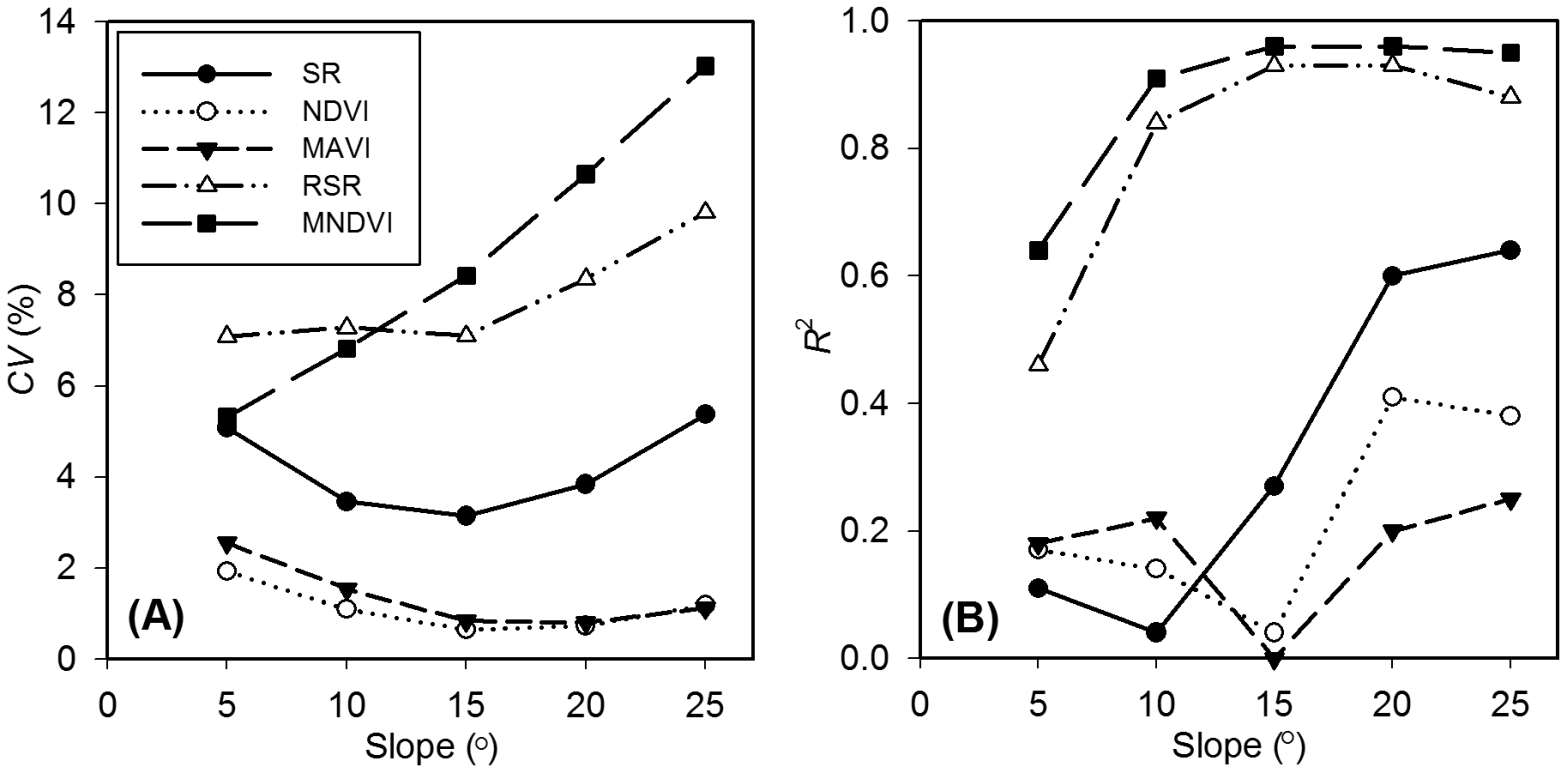
Effects of slope variations on different vegetation indices. Note: (A) the coefficient of variation (*CV*) of each vegetation index varies with slopes, (B) the *R* ^2^ values of linear correlations between vegetation indices and the cosine of the incidence angle vary with slopes. The *CV* values of MNDVI vary from 5.32% to 13.02% corresponding to the slopes from 5° to 25°, which shows the largest topographical noise among all the selected vegetation indices. RSR has the second largest *CV* values ranging from 7.09% to 9.81%. SR presents a medium *CV* values in the range from 3.14% to 5.37%. The *CV* values are quite small for NDVI and MAVI ranging from 0.64% to 1.92% and from 0.80% to 2.56%, respectively, implying that NDVI and MAVI can remove much of topographic noise through expressing in band-ratio form. The conclusions based on *R* ^2^ are also similar.

Figure 9 depicts quantitatively the topographic effects on the selected VIs. The *CV* values of MNDVI vary from 5.32% to 13.02% corresponding to the slopes from 5° to 25°, showing the largest topographical noise among all the selected VIs. RSR has the second largest *CV* values ranging from 7.09% to 9.81%. SR presents medium *CV* values in the range from 3.14% to 5.37%. The *CV* values are quite small for NDVI and MAVI ranging from 0.64% to 1.92% and from 0.80% to 2.56%, respectively, implying that NDVI and MAVI can remove much of the topographic noise through expressing in band-ratio form.

In general, the *R* ^2^ values of linear correlations between VI and the cosine of the incidence angle (cos*i*) increase as the slope increases (Figure 9). The *R* ^2^ values are in the range from 0.46 to 0.93 for RSR and from 0.64 to 0.96 for MNDVI in the study area, indicating that VIs not based on band ratios are influenced strongly by topographic variations. When the slope is above 20°, the largest *R* ^2^ values are 0.64 for SR and 0.41 for NDVI. The results demonstrate that even VIs based on band-ratios still include significant topographic effects on steep slopes, confirming the findings of Burgess et al. [40] and Kusaka and Sakane [41]. When the slope is 25°, the *R* ^2^ value of MAVI is 0.25, which proves that MAVI performs rather effectively in removing the effects of slopes. SR is more sensitive to topographic variations on steep slopes than NDVI and MAVI, which may be due to the relatively different topographic effects on the red and NIR bands. Therefore, the topographic effects should be removed before the applications of VIs that are not based on band ratios, even when slopes are small. The topographic effects on VIs expressed in band-ratio form can usually be ignored on small slopes, but careful topographic corrections are needed when they are used over the rugged surface.

## Conclusions

In this study, we develop a new three-band moisture adjusted vegetation index (MAVI). Its performance is evaluated against commonly used two-band VIs (NDVI, SR, SAVI, MSAVI, OSAVI) and three-band VIs (MDNVI, RSR) with field measurements made in two forest and two grassland areas in China. The reflectance data simulated by the 4-Scale model is also use to investigate the background reflectance effects of MAVI on forest canopy LAI retrieval. The following conclusions can be drawn from this study:

(1) MAVI is suitable for retrieving LAI using remote sensing images. It produces a higher *R* ^2^, smaller normalized RMSE of retrieved LAI compared with two-band VIs in both forest and grassland areas. The superior performance of MAVI over two-band VIs is mainly due to its integration of the signals from the red, NIR, and SWIR bands sensitive to the greenness, chlorophyll, and water content of the vegetation.

(2) MAVI can reduce the background reflectance effects on forest canopy LAI retrieval as effectively as RSR and MNDVI. It outperformed RSR and MNDVI for retrieving LAI in the four study areas without the need for inputting the maximum and minimum SWIR values, which are notoriously difficult to determine.

(3) Topography strongly affects VIs that are not based on band ratios, such as RSR and MNDVI. Since MAVI is expressed as ratios between spectral bands, it can greatly reduce the noise caused by topographical variations, which makes it suitable for application in mountainous area.

In this study, validation shows the robustness of MAVI in retrieving LAI of forests and grasses. Because the SWIR reflectance is also affected by the wetness of soils, especially when vegetation density is low, the robustness of MAVI may need further validation using more data from other ecosystems.
